# (3*S*,4*S*,5*R*)-4-Hydr­oxy-3-methyl-5-[(2*S*,3*R*)-3-methyl­pent-4-en-2-yl]-4,5-dihydro­furan-2(3*H*)-one

**DOI:** 10.1107/S1600536808042414

**Published:** 2008-12-17

**Authors:** Annika Gille, Dieter Bläser, Roland Boese, Hans Preut, Martin Hiersemann

**Affiliations:** a Fakultät Chemie, Technische Universität Dortmund, Otto-Hahn-Strasse 6, 44221 Dortmund, Germany; b Fachbereich Chemie, Universität Duisburg-Essen, Campus Essen UniversitätsStrasse 7, 45117 Essen, Germany

## Abstract

The relative configuration of the title compound, C_11_H_18_O_3_, was corroborated by single-crystal X-ray diffraction analysis. In the crystal, mol­ecules are linked *via* a O—H⋯O hydrogen bond and a chain of mol­ecules is formed along [010].

## Related literature

For further synthetic details, see: Abraham, Körner & Hierse­mann (2004 ▶); Abraham, Körner *et al.* (2004 ▶); Evans *et al.* (1981 ▶, 1999 ▶); Körner & Hiersemann (2006 ▶, 2007 ▶); Mitsunobu (1981 ▶); Mitsunobu & Yamada (1967 ▶); Mitsunobu *et al.* (1967 ▶); Otera *et al.* (1992 ▶); Pollex & Hiersemann (2005 ▶).
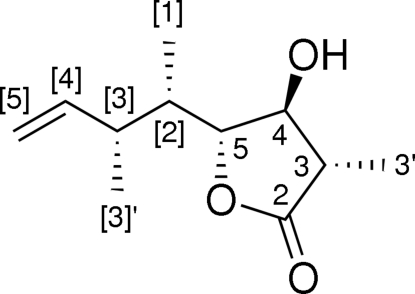


## Experimental

### 

#### Crystal data

C_11_H_18_O_3_
*M* *_r_* = 198.25
Monoclinic, 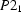

*a* = 7.604 (2) Å
*b* = 6.574 (2) Å
*c* = 11.323 (4) Å
β = 91.211 (7)°
*V* = 565.9 (3) Å^3^
*Z* = 2
Mo *K*α radiation
μ = 0.08 mm^−1^
*T* = 173 (2) K
0.35 × 0.10 × 0.07 mm

#### Data collection

Siemens SMART three-axis goniometer with APEXII area-detector diffractometer
Absorption correction: none
7868 measured reflections
1507 independent reflections
1076 reflections with *I* > 2σ(*I*)
*R*_int_ = 0.131

#### Refinement

*R*[*F* ^2^ > 2σ(*F* ^2^)] = 0.054
*wR*(*F* ^2^) = 0.098
*S* = 0.98
1507 reflections
132 parameters
1 restraint
H-atom parameters constrained
Δρ_max_ = 0.19 e Å^−3^
Δρ_min_ = −0.19 e Å^−3^

### 

Data collection: *APEX2* (Bruker, 2004 ▶); cell refinement: *SAINT* (Bruker, 2004 ▶); data reduction: *SAINT*; program(s) used to solve structure: *SHELXS97* (Sheldrick, 2008 ▶); program(s) used to refine structure: *SHELXL97* (Sheldrick, 2008 ▶); molecular graphics: *SHELXTL-Plus* (Sheldrick, 2008 ▶); software used to prepare material for publication: *SHELXL97* and *PLATON* (Spek, 2003 ▶).

## Supplementary Material

Crystal structure: contains datablocks I, global. DOI: 10.1107/S1600536808042414/hb2861sup1.cif

Structure factors: contains datablocks I. DOI: 10.1107/S1600536808042414/hb2861Isup2.hkl

Additional supplementary materials:  crystallographic information; 3D view; checkCIF report

## Figures and Tables

**Table 1:** Hydrogen-bond geometry (Å, °)

*D*—H⋯*A*	*D*—H	H⋯*A*	*D*⋯*A*	*D*—H⋯*A*
O2—H2⋯O3^i^	0.84	2.02	2.830 (3)	163

Symmetry code: (i) 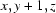.

**Fig. 1.**
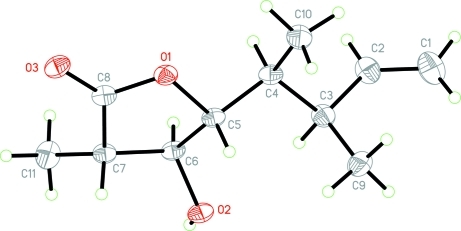
: The molecular structure of (I) with displacement ellipsoids for the non-hydrogen atoms shown at the 30% probability level.

## supplementary crystallographic information

### Comment

The title compound, (I), was synthesized using a catalytic asymmetric Claisen rearrangement (Abraham, Körner *et al.*, 2004; Abraham, Körner & Hiersemann, 2004; Pollex & Hiersemann, 2005; Körner & Hiersemann, 2006, 2007), a diastereoselective reduction with K-Selectride (Körner & Hiersemann, 2006, 2007), a Mitsunobu reaction (Mitsunobu & Yamada, 1967; Mitsunobu *et al.*, 1967; Mitsunobu, 1981) and an Evans aldol addition (Evans *et al.*, 1981). In order to verify the relative configuration of the obtained aldol adduct, 4-(*tert*-butyldimethylsilyloxy)-3-hydroxy-2,5,6-trimethyloct-7-enoyl)-4-isopropyloxazolidin-2-one, (II), a γ-lactone, (I), was prepared by removal of the silyl protecting group (Otera *et al.*, 1992) and subsequent *in situ* lactonization. Fig. 1 depicts the structure of the isolated diastereomer (I). The configuration of the chiral C atoms in (I) can be attributed to the stereochemical course of the Evans aldol addition (C3 *S* and C4 *S*), the diastereoselective reduction with K-Selectride (C5 *S*) followed by Mitsunobu reaction (C5 *R*), and the catalytic asymmetric Claisen rearrangement (C[2] *S* and C[3] *R*) using the chiral Lewis acid [Cu{(*S*,*S*)-*tert*-Butyl-box}](H_2_O)_2_(SbF_6_)_2_ (Evans *et al.*, 1999).

In the crystal, an O—H···O hydrogen bond (Table 1) links the molecules into chains propagating in [010].

### Experimental

The title compound, (I), was synthesized from the corresponding *syn*-aldol adduct, (II), using tetrabutylammonium fluoride (TBAF) in the presence of acetic acid (Otera *et al.*, 1992) for the removal of the silyl protecting group. The subsequent lactonization proceeded *in situ*.

To a solution of diastereomerically pure (II) (66 mg, 0.15 mmol, 1.0 eq) in tetrahydrofuran (0.8 ml) was added acetic acid (0.9 µl, 0.015 mmol, 0.1 eq) in tetrahydrofuran (0.1 ml) and TBAF (1 *M* in tetrahydrofuran, 0.225 ml, 1.5 eq) at 273 K. After stirring for 20 min at 273 K, the mixture was allowed to warm to room temperature. After stirring the reaction mixture for 4.5 h at room temperature, the reaction was quenched by the addition of sat. aqueous NH_4_Cl solution. The phases were separated, and the aqueous phase was extracted with CH_2_Cl_2_ (3 × 5 ml). The combined organic layers were dried (MgSO_4_) and concentrated under reduced pressure. Flash chromatography (isohexane/ethyl acetate 20/1 to 10/1 to 5/1) afforded (I) as a single diastereomer in quantitative yield (29.6 mg, 0.15 mmol) as colourless crystals.  Colourless needles of (I) were obtained by vapor diffusion recrystallization technique from isohexane and ethyl acetate: mp 361 K; R*_f_* 0.35 (cyclohexane/ethyl acetate 2/1); ^1^H NMR (CDCl_3_, 400 MHz, δ): 0.92 (d, *J* = 6.8 Hz, 3H), 1.05 (d, *J* = 6.8 Hz, 3H), 1.31 (d, *J* = 7.1 Hz, 3H), 1.73 (dqd, *J* = 6.8, 6.8, 3.6 Hz, 1H), 2.14 (br. s, 1 H), 2.33 (apparent sex, *J* = 7.7 Hz, 1H), 2.59 (dq, *J* = 8.6, 7.1 Hz, 1H), 3.91 (dd, *J* = 8.6 Hz, 1H), 4.20 (dd, *J* = 8.6, 3.6 Hz, 1H), 5.04 (d, ^3^*J*(*Z*) = 10.0 Hz, 1H), 5.05 (d, ^3^*J*(*E*) = 17.8 Hz, 1H), 5.73 (ddd, ^3^*J*(*E*) = 17.8 Hz, ^3^*J*(*Z*) = 10.0 Hz, ^3^*J* = 7.7 Hz, 1H); ^13^C NMR (CDCl_3_, 100 MHz, δ): 10.7 (CH_3_), 12.8 (CH_3_), 17.2 (CH_3_), 39.4 (CH), 41.0 (CH), 44.2 (CH), 77.2 (CH), 85.2 (CH), 115.1 (CH_2_), 143.0 (CH), 176.7 (C); IR (cm^-1^): 3405(*br*,*s*) (ν O—H, OH in H-bridges), 3090(w) 3005(w) (ν C—H, olefin), 2965(*s*) 2935(*m*) 2900(*m*) 2855(*w*) (ν_as,s_ C—H, CH_2_, CH_3_, CH), 1740(*s*) (ν C=O, lactone), 1640(*w*) (ν C=C), 1460(*s*) (δ_as_ C—H, CH_3_, CH_2_), 1380(*s*) (δ_s_ C—H, CH_3_); Anal. Calcd. for C_11_H_18_O_3_: C, 66.6; H, 9.2; Found: C, 66.7; H, 9.3; [α]_D_^20^ +61.4 (c 0.487, CHCl_3_).

### Refinement

The H atoms were geometrically placed (C—H = 0.95–1.00Å, O—H = 0.84Å) and refined as riding with U_iso_(H) = 1.2U_eq_(C,O) or 1.5U_eq_(methyl C).

### Crystal data

**Table:**

C_11_H_18_O_3_	*F*(000) = 216
*M**_r_* = 198.25	*D*_x_ = 1.163 Mg m^−^^3^
Monoclinic, *P*2_1_	Mo *K*α radiation, λ = 0.71073 Å
Hall symbol: P 2yb	Cell parameters from 1365 reflections
*a* = 7.604 (2) Å	θ = 2.7–26.3°
*b* = 6.574 (2) Å	µ = 0.08 mm^−^^1^
*c* = 11.323 (4) Å	*T* = 173 K
β = 91.211 (7)°	Needle, colourless
*V* = 565.9 (3) Å^3^	0.35 × 0.10 × 0.07 mm
*Z* = 2	

### Data collection

**Table:**

Siemens SMART three-axis goniometer with APEXII area-detector system diffractometer	1076 reflections with *I* > 2σ(*I*)
Radiation source: fine-focus sealed tube	*R*_int_ = 0.131
graphite	θ_max_ = 28.3°, θ_min_ = 1.8°
Detector resolution: 512 pixels mm^-1^	*h* = −9→10
Data collection strategy APEX 2/COSMO scans	*k* = −8→8
7868 measured reflections	*l* = −15→14
1507 independent reflections	

### Refinement

**Table:**

Refinement on *F*^2^	Secondary atom site location: difference Fourier map
Least-squares matrix: full	Hydrogen site location: inferred from neighbouring sites
*R*[*F*^2^ > 2σ(*F*^2^)] = 0.054	H-atom parameters constrained
*wR*(*F*^2^) = 0.098	*w* = 1/[σ^2^(*F*_o_^2^) + (0.0279*P*)^2^] where *P* = (*F*_o_^2^ + 2*F*_c_^2^)/3
*S* = 0.98	(Δ/σ)_max_ < 0.001
1507 reflections	Δρ_max_ = 0.19 e Å^−^^3^
132 parameters	Δρ_min_ = −0.19 e Å^−^^3^
1 restraint	Extinction correction: *SHELXL97* (Sheldrick, 2008), Fc^*^=kFc[1+0.001xFc^2^λ^3^/sin(2θ)]^-1/4^
Primary atom site location: structure-invariant direct methods	Extinction coefficient: 0.077 (11)

### Special details

**Table:**

Geometry. All e.s.d.'s (except the e.s.d. in the dihedral angle between two l.s. planes) are estimated using the full covariance matrix. The cell e.s.d.'s are taken into account individually in the estimation of e.s.d.'s in distances, angles and torsion angles; correlations between e.s.d.'s in cell parameters are only used when they are defined by crystal symmetry. An approximate (isotropic) treatment of cell e.s.d.'s is used for estimating e.s.d.'s involving l.s. planes.
Refinement. Refinement of *F*^2^ against ALL reflections. The weighted *R*-factor *wR* and goodness of fit *S* are based on *F*^2^, conventional *R*-factors *R* are based on *F*, with *F* set to zero for negative *F*^2^. The threshold expression of *F*^2^ > σ(*F*^2^) is used only for calculating *R*-factors(gt) *etc*. and is not relevant to the choice of reflections for refinement. *R*-factors based on *F*^2^ are statistically about twice as large as those based on *F*, and *R*- factors based on ALL data will be even larger.

### Fractional atomic coordinates and isotropic or equivalent isotropic displacement parameters (Å^2^)

**Table:**

	*x*	*y*	*z*	*U*_iso_*/*U*_eq_	
O1	0.2255 (2)	0.2085 (3)	0.91883 (16)	0.0351 (5)	
O2	0.1493 (2)	0.7353 (3)	0.96072 (16)	0.0382 (5)	
H2	0.2076	0.8318	0.9904	0.057*	
O3	0.2839 (2)	0.0670 (3)	1.09375 (17)	0.0408 (5)	
C1	0.3186 (5)	0.7143 (7)	0.4846 (3)	0.0700 (11)	
H1A	0.1994	0.7345	0.4607	0.084*	
H1B	0.4096	0.7302	0.4292	0.084*	
C2	0.3570 (4)	0.6651 (5)	0.5929 (3)	0.0471 (8)	
H2A	0.4783	0.6469	0.6118	0.057*	
C3	0.2316 (4)	0.6337 (4)	0.6919 (2)	0.0381 (7)	
H3	0.2615	0.7378	0.7535	0.046*	
C4	0.2658 (4)	0.4224 (4)	0.7492 (2)	0.0352 (7)	
H4	0.3959	0.4082	0.7615	0.042*	
C5	0.1827 (3)	0.4084 (4)	0.8699 (2)	0.0305 (6)	
H5	0.0522	0.4226	0.8610	0.037*	
C6	0.2512 (3)	0.5554 (4)	0.9651 (2)	0.0309 (6)	
H6	0.3778	0.5877	0.9522	0.037*	
C7	0.2319 (4)	0.4339 (4)	1.0786 (2)	0.0324 (6)	
H7	0.1086	0.4522	1.1059	0.039*	
C8	0.2500 (3)	0.2207 (4)	1.0367 (2)	0.0327 (6)	
C9	0.0401 (4)	0.6683 (5)	0.6558 (3)	0.0513 (8)	
H9A	0.0246	0.8089	0.6288	0.077*	
H9B	−0.0348	0.6434	0.7236	0.077*	
H9C	0.0072	0.5748	0.5916	0.077*	
C10	0.2046 (4)	0.2460 (5)	0.6698 (3)	0.0468 (8)	
H10A	0.2487	0.1173	0.7025	0.070*	
H10B	0.2503	0.2650	0.5902	0.070*	
H10C	0.0758	0.2429	0.6657	0.070*	
C11	0.3561 (4)	0.4916 (5)	1.1806 (3)	0.0477 (8)	
H11A	0.3352	0.4020	1.2481	0.071*	
H11B	0.3347	0.6331	1.2035	0.071*	
H11C	0.4781	0.4767	1.1558	0.071*	

### Atomic displacement parameters (Å^2^)

**Table:**

	*U*^11^	*U*^22^	*U*^33^	*U*^12^	*U*^13^	*U*^23^
O1	0.0416 (11)	0.0232 (11)	0.0406 (11)	0.0024 (9)	0.0034 (8)	−0.0022 (8)
O2	0.0413 (11)	0.0230 (10)	0.0504 (12)	0.0052 (9)	0.0008 (9)	−0.0046 (9)
O3	0.0428 (12)	0.0276 (11)	0.0520 (13)	0.0029 (9)	0.0009 (10)	0.0033 (9)
C1	0.078 (2)	0.083 (3)	0.050 (2)	−0.008 (2)	0.0124 (17)	0.006 (2)
C2	0.0516 (19)	0.0459 (19)	0.0439 (19)	−0.0065 (15)	0.0038 (15)	−0.0007 (14)
C3	0.0457 (18)	0.0296 (15)	0.0391 (17)	−0.0013 (13)	0.0026 (14)	−0.0019 (12)
C4	0.0367 (16)	0.0273 (15)	0.0418 (16)	0.0022 (13)	0.0027 (12)	−0.0043 (13)
C5	0.0327 (14)	0.0184 (13)	0.0403 (16)	−0.0005 (12)	0.0005 (12)	−0.0025 (12)
C6	0.0294 (15)	0.0194 (14)	0.0441 (16)	−0.0010 (11)	0.0024 (12)	−0.0012 (12)
C7	0.0328 (15)	0.0250 (14)	0.0396 (16)	−0.0016 (12)	0.0010 (12)	−0.0060 (12)
C8	0.0275 (14)	0.0268 (14)	0.0440 (17)	−0.0030 (12)	0.0027 (12)	−0.0020 (14)
C9	0.057 (2)	0.0446 (19)	0.0527 (19)	0.0102 (16)	0.0027 (15)	0.0093 (14)
C10	0.066 (2)	0.0294 (16)	0.0447 (17)	0.0034 (16)	−0.0029 (15)	−0.0096 (14)
C11	0.054 (2)	0.0397 (17)	0.0489 (19)	−0.0075 (15)	−0.0091 (15)	0.0000 (14)

### Geometric parameters (Å, °)

**Table:**

O1—C8	1.347 (3)	C5—C6	1.531 (4)
O1—C5	1.460 (3)	C5—H5	1.0000
O2—C6	1.414 (3)	C6—C7	1.522 (4)
O2—H2	0.8400	C6—H6	1.0000
O3—C8	1.223 (3)	C7—C8	1.487 (4)
C1—C2	1.296 (4)	C7—C11	1.524 (4)
C1—H1A	0.9500	C7—H7	1.0000
C1—H1B	0.9500	C9—H9A	0.9800
C2—C3	1.501 (4)	C9—H9B	0.9800
C2—H2A	0.9500	C9—H9C	0.9800
C3—C9	1.521 (4)	C10—H10A	0.9800
C3—C4	1.553 (4)	C10—H10B	0.9800
C3—H3	1.0000	C10—H10C	0.9800
C4—C5	1.520 (4)	C11—H11A	0.9800
C4—C10	1.534 (4)	C11—H11B	0.9800
C4—H4	1.0000	C11—H11C	0.9800
			
C8—O1—C5	110.4 (2)	C7—C6—H6	110.2
C6—O2—H2	109.5	C5—C6—H6	110.2
C2—C1—H1A	120.0	C8—C7—C6	102.4 (2)
C2—C1—H1B	120.0	C8—C7—C11	114.6 (2)
H1A—C1—H1B	120.0	C6—C7—C11	116.1 (2)
C1—C2—C3	127.4 (3)	C8—C7—H7	107.8
C1—C2—H2A	116.3	C6—C7—H7	107.8
C3—C2—H2A	116.3	C11—C7—H7	107.8
C2—C3—C9	113.5 (2)	O3—C8—O1	119.8 (3)
C2—C3—C4	109.4 (2)	O3—C8—C7	129.1 (2)
C9—C3—C4	113.4 (2)	O1—C8—C7	111.1 (2)
C2—C3—H3	106.7	C3—C9—H9A	109.5
C9—C3—H3	106.7	C3—C9—H9B	109.5
C4—C3—H3	106.7	H9A—C9—H9B	109.5
C5—C4—C10	110.8 (2)	C3—C9—H9C	109.5
C5—C4—C3	111.1 (2)	H9A—C9—H9C	109.5
C10—C4—C3	112.7 (2)	H9B—C9—H9C	109.5
C5—C4—H4	107.3	C4—C10—H10A	109.5
C10—C4—H4	107.3	C4—C10—H10B	109.5
C3—C4—H4	107.3	H10A—C10—H10B	109.5
O1—C5—C4	107.56 (19)	C4—C10—H10C	109.5
O1—C5—C6	103.3 (2)	H10A—C10—H10C	109.5
C4—C5—C6	117.0 (2)	H10B—C10—H10C	109.5
O1—C5—H5	109.5	C7—C11—H11A	109.5
C4—C5—H5	109.5	C7—C11—H11B	109.5
C6—C5—H5	109.5	H11A—C11—H11B	109.5
O2—C6—C7	113.9 (2)	C7—C11—H11C	109.5
O2—C6—C5	109.0 (2)	H11A—C11—H11C	109.5
C7—C6—C5	103.1 (2)	H11B—C11—H11C	109.5
O2—C6—H6	110.2		
			
C1—C2—C3—C9	−0.8 (5)	C4—C5—C6—O2	90.6 (3)
C1—C2—C3—C4	126.9 (4)	O1—C5—C6—C7	−30.1 (2)
C2—C3—C4—C5	163.2 (2)	C4—C5—C6—C7	−148.1 (2)
C9—C3—C4—C5	−69.1 (3)	O2—C6—C7—C8	146.5 (2)
C2—C3—C4—C10	−71.8 (3)	C5—C6—C7—C8	28.6 (3)
C9—C3—C4—C10	55.9 (3)	O2—C6—C7—C11	−88.0 (3)
C8—O1—C5—C4	144.9 (2)	C5—C6—C7—C11	154.1 (2)
C8—O1—C5—C6	20.6 (2)	C5—O1—C8—O3	178.6 (2)
C10—C4—C5—O1	55.7 (3)	C5—O1—C8—C7	−2.0 (3)
C3—C4—C5—O1	−178.3 (2)	C6—C7—C8—O3	161.8 (3)
C10—C4—C5—C6	171.3 (2)	C11—C7—C8—O3	35.3 (4)
C3—C4—C5—C6	−62.6 (3)	C6—C7—C8—O1	−17.5 (3)
O1—C5—C6—O2	−151.49 (19)	C11—C7—C8—O1	−144.0 (2)

### Hydrogen-bond geometry (Å, °)

**Table:**

*D*—H···*A*	*D*—H	H···*A*	*D*···*A*	*D*—H···*A*
O2—H2···O3^i^	0.84	2.02	2.830 (3)	163

Symmetry codes: (i) *x*, *y*+1, *z*.
